# Assessment of Lithium, Macro- and Microelements in Water, Soil and Plant Samples from Karst Areas in Romania

**DOI:** 10.3390/ma14144002

**Published:** 2021-07-17

**Authors:** Anamaria Iulia Török, Ana Moldovan, Erika Andrea Levei, Oana Cadar, Claudiu Tănăselia, Oana Teodora Moldovan

**Affiliations:** 1 INCDO-INOE 2000, Research Institute for Analytical Instrumentation, 67 Donath, 400293 Cluj-Napoca, Romania; iulia.torok@icia.ro (A.I.T.); erika.levei@icia.ro (E.A.L.); oana.cadar@icia.ro (O.C.); claudiu.tanaselia@icia.ro (C.T.); 2 Faculty of Materials and Environmental Engineering, Technical University, 103-105 Muncii Boulevard, 400641 Cluj-Napoca, Romania; 3 Cluj Department, Emil Racovita Institute of Speleology, 5 Clinicilor, 400006 Cluj-Napoca, Romania; oanamol35@gmail.com

**Keywords:** lithium, groundwater, karst, plants, *Lolium*, *Urtica*, *Mentha*, bioconcentration factor

## Abstract

Lithium is a critical element for the modern society due to its uses in various industrial sectors. Despite its unequal distribution in the environment, Li occurrence in Romania was scarcely studied. In this study a versatile measurement method using ICP-MS technique was optimized for the determination of Li from various matrixes. Water, soil, and plant samples were collected from two important karst areas in the Dobrogea and Banat regions, Romania. The Li content was analyzed together with other macro- and microelement contents to find the relationship between the concentration of elements and their effect on the plants’ Li uptake. In Dobrogea region, half of the studied waters had high Li concentration, ranging between 3.00 and 12.2 μg/L in the case of water and between 0.88 and 11.1 mg/kg DW in the case of plants, while the Li content in the soil samples were slightly comparable (from 9.85 to 11.3 mg/kg DW). In the Banat region, the concentration of Li was lower than in Dobrogea (1.40–1.46 μg/L in water, 6.50–9.12 mg/kg DW in soil, and 0.19–0.45 mg/kg DW in plants). Despite the high Li contents in soil, the Li was mostly unavailable for plants uptake and bioaccumulation.

## 1. Introduction

Lithium (Li) is one of the most “critical” metals for modern society used in many fields such as manufacturing electronic devices, glass and ceramics, as well in medical and cosmetics products [1]. It is the 30th most abundant element in the upper continent crust with similar abundance to Pb and Cu, naturally occurring in waters, soils, rocks, and minerals (such as lithium micas, amblygonite, petalite, lepidolite, spodumene, eucryptite) [2,3,4]. The average concentrations in shale and granitic rocks are 5 to 10 times higher than those in carbonate-based rocks [3]. The main source of Li in the environment is the weathering of minerals, its abundance depending on the lithology, topography, and hydrology.

The biochemical role of Li in the lifecycle of living organisms is unclear, though is considered to have a potential role as a micronutrient [5]. It is adsorbed by sodium channels in the intestines and it can be detected in the serum, saliva, and urine [2]. Microdoses of Li intake through drinking water may have antisuicidal, mood-stabilizing, antidepressive and antimanic effects. Moreover, the dietary Li was found to have a beneficial role in the prevention of dementia and Alzheimer’s diseases [2,6,7]. However, the biochemical mechanisms are still unelucidated. Some studies report that the positive association of Li consumption is overestimated or there is a lack of data on the long-term effects of Li intake [6]. Overall, more investigation needs to be conducted to verify the hypothesis that Li has a potentially protective and beneficial impact on human health and to determine the role of the naturally occurring Li in different regions. In addition, the identification of potential dietary Li sources is relevant for future research areas [6].

Drinking water, grains, or vegetables can be a major Li source for humans [5]. A recent study reported that the Li content in food samples ranges between <0.001 and 4.238 mg/kg, with vegetables containing the highest concentration followed by milk, cheese, meat, and oil products [8]. Naturally, the Li concentration ranges between 7 and 200 mg/kg in soil, between 1 and 10 µg/L in surface water, and around 20 µg/L in seawater [9]. European survey studies reported Li concentrations in bottled and tap water ranging between 2.65 and 14.9 µg/L [9]. Therefore, the estimation of Li daily intake can be different depending on the availability in the environment and food products [10].

Karst aquifers are an important resource of water, providing 50% of the world‘s drinking water [11]. The sources of Li in a karst system are mainly represented by silicate dissolution, atmospheric input via rainfall and sea-spray aerosol, followed by carbonate dissolution [12]. Generally, the drinking water Li concentrations can vary from <1 to 219 µg/L [2,13,14]. Presently, there are no recommendations or regulatory thresholds for Li concentration in drinking water.

Li concentrations can be determined using different analytical techniques such as atomic absorption spectrometry (AAS), inductively coupled plasma atomic emission spectrometry (ICP-AES), and inductively coupled plasma mass spectrometry (ICP-MS) or by ion-selective electrodes [15]. Of these, the ICP-MS technique is a sensitive method that was frequently used for determination of elements from various types of samples, such as water, wine, saliva, blood, urine, plant, sediment, and rocks [2].

The identification and assessment of Li resources is a key step for understanding lithium metallogeny. Moreover, the identification of the Li uptake rate is another important factor, which can contribute to a better understanding of the role of Li in the living organisms. In this study, a versatile quantitative ICP-MS method for Li determination in three different sample matrixes (water, soil, and plant samples) was optimized and the relationship between the Li, macroelements (Na, Mg, Al, K, Ca, Fe, Mn) and microelements (Cr, Co, Ni, Cu, Zn, Pb, Sr, Ba, V, As, Sr, Cd, Pb) concentration in water, soil, and plants (ryegrass—*Lolium* sp., nettles—*Urtica* sp., and mint—*Mentha* sp.) in two important karst areas from Romania were assessed to display the divergence of the Li content in the samples and to find a connection between Li concentration in plants compared to soil and water.

## 2. Materials and Methods

### 2.1. Description of the Study Areas and Sampling Campaign

Groundwater, soil, and plant samples were collected in September 2020 from six sampling points located in: rural areas of Dobrogea region, south-eastern Romania (GWR27, GWR28, GWR29, and GWR30) and a protected area of Banat region, south-western Romania (GN and GWR6). Details on sampling sites, geology, soil type, and type of plants collected are given in Table 1. Dobrogea is characterized by faulted limestones, dolomitic limestones, and thick layers of carbonate successions, the main groundwater type for the sampling sites being represented by local or discontinuous aquifers in fissured rocks [16]. The parental bedrock materials of the study area from Banat region mainly includes Barremian–Aptian limestones, while the groundwater sources are related to extended aquifers (GN) and local or discontinuous aquifers (GWR6). The predominant soil type in Dobrogea is cambic-chernozem and chernozem, while in southern Banat region is cambisol with rendzinas and rocky areas in the zones with karst [17,18].

In each sampling site, 1 L of groundwater was collected from springs in precleaned polyethylene bottles and kept at 4 °C until analysis. A composite soil sample (1.5–2 kg) was obtained by mixing 5 subsamples collected from an area of 50 m^2^ in the vicinity of each spring, from a depth of 0–30 cm and by using a stainless-steel shovel. Depending on their presence near each spring, 3 to 5 plants were collected with a Teflon coated knife. The soil and plant samples were stored in paper bags during transport to laboratory.

### 2.2. Sample Preparation

For the macro- and microelements determination, the water samples were filtered through 0.45 μm cellulose acetate membrane filters and acidulated with 65% HNO_3_ to pH < 2. Soil samples were oven dried at 105 °C, ground to pass through a 2 mm size sieve, and stored in closed polyethylene bags. An amount of 3 g of soil sample were digested with a 1:3 *v/v* mixture of 37% HCl and 65% HNO_3_ [19]. The digested soil samples were transferred into 100 mL volumetric flasks and diluted with ultrapure water (Elga Veolia, High Wycombe, UK). Plant samples were washed with distilled water, oven dried at 65 °C, and ground by an agate mortar and passed through a 200 µm mesh sieve to obtain a homogenized powder. An amount of 2 g of plant material was digested with 5 mL 65% HNO_3_ and 2 mL 30% H_2_O_2_. The digested samples were diluted with ultrapure water to a final volume of 25 mL [19]. The measured metal concentrations are expressed as mg/kg dry weight (DW).

All reagents were of analytical purity and were used without further purification. Ultrapure deionized water from a water purification system (Elga Veolia, High Wycombe, UK) and ultra-pure 60% HNO_3_ (Merck, Darmstadt, Germany) were used for all dilutions and to prepare the calibration standard solutions. All the labware prior to use were cleaned by soaking for 24 h in 10% HNO_3_ and then rinsed with ultra-pure water.

Analysis of Na, Mg, K, Ca, and Fe was performed using 5300 Optima DV (Perkin–Elmer, Waltham, MA, USA) Inductively Coupled Plasma Optical Emission Spectrometer (ICP-OES) while Li, Al, V, Cr, Mn, Co, Ni, Cu, Zn, As, Sr, Cd, Ba, and Pb with ELAN DRC II (Perkin–Elmer, Waltham, MA, USA) Inductively Coupled Plasma Quadrupole Mass Spectrometer (ICP-MS). Certified multielement ICP Standard 3 with a concentration of 10 µg/mL (Perkin–Elmer, Waltham, MA, USA) was used to prepare calibration standards. Calibration was linear with correlation coefficients (R^2^) above 0.9996.

For the Li measurement, the ICP-MS equipped with a Meinhard type nebulizer containing cyclonic spray chamber and was used under the continuous nebulization conditions. Before each run, the spectrometer was optimized for best signal/noise ratio: an indium solution was fed into the instrument and its signal maximized, while the background was kept below 2 counts per second (cps). To avoid any potential interferences, oxides and double charged ions were kept under 3% by carefully tunning instrument parameters and oxides and double charged ion formation was monitored by measuring Ce/CeO and Ba/Ba^2+^ ratios from a certified solution at the beginning of each sample batch. For the quality assurance of results, calibration standards, procedural blank, and triplicate sample measurements were used.

To evaluate the performances of the Li determination in water, soil, and plant samples, the limit of detection (LOD), limit of quantification (LOQ), repeatability, and reproducibility were assessed. The LOD and LOQ were calculated as 3 and 10 times of the standard deviation of the Li intensity of a blank solution containing ultrapure water and 5% *v*/*v* HNO_3_ [20]. The following certified reference material (CRMs) were analyzed to check the accuracy of the Li measurements: 1643f NIST (National Institute of Standards and Technology, Gaithersburg, MD, USA) for water, SQC001 NIST SRM Loam Clay (Sigma–Aldrich RTC, USA) for soil and NIM-GWB 10019 Apple-Trace elements (Institute of Geophysical and Geochemical Exploration, Langfang City, China) for plants.

### 2.3. Multivariate Statistics

Principal component analysis (PCA) is a powerful tool to explain a variance of interrelated variables for reducing the dimensionality of the data set [21]. The main characteristics of the PCA is to reduce a large number of variables into a new set of reduced variables based on their mutual dependence and to identify the difference among the three matrix samples metal concentration and to show a correlation among the variables. In the present study, the PCA was performed using OriginLab (2020b) software based on the eigenvalues of the correlation matrix on standardized data and was used to maximize the variation expressed by the principal components. Only PC’s with eigenvalues >1 were retained. Also, OriginLab (2020b) software was used to present the soil samples date in heat map associated with the agglomerative hierarchical cluster analysis (HCA) using the HeatMapDendogram Apps with Pearson correlation settings.

### 2.4. Bioconcentration Factor

The bioconcentration factor (BCF) was computed to determine the plant samples macro- and microelement accumulation capacity in the different sampling locations. The BCF was calculated as the ratio between the metal concentration in the individual plant and the metal concentration in the soil sample, according to the suggestion of Gajić et al. [22].

## 3. Results

### 3.1. ICP-MS Parameters Optimization

To optimize the Li determination from three different sample matrixes using ICP-MS method, some basic characteristics were taken into consideration such as, the sample matrix type (water, soil, and plant samples), the analyte concentration level and range, presence of interfering substances (organic substance content, which was eliminated through samples’ mineralization), requirements for the procedure detection limits and quantification based on blank samples, determination of the repeatability based on the standard deviation for data series, and criteria for the use of reference material to describe the accuracy of the analytical procedure.

Li had no interelement spectral interferences. The equipment (ICP-MS, Elan DRC II, Perkin–Elmer) parameters and conditions for the Li determination analytical procedure are given in the Table 2.

**Figure 1:**
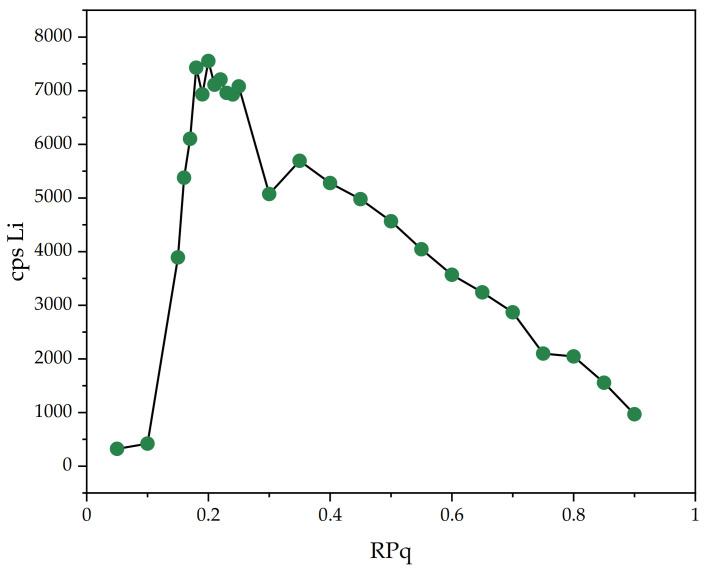
Plot of Li cps in contrast with RPq during method optimization using ICP-MS spectrometer (Perkin–Elmer). A value of RPq = 0.22 was used for all Li measurements.

The dynamic reaction cell (DRC) was used in rf-only mode (vented, no gas) and rejection parameter q (RPq) of the cell was tuned for maximum Li signal intensity by continuously measuring the signal intensity from the same sample, while varying the RPq value. The second rejection parameter (RPa) was set to its default value (zero) and since its effect on the ion passage through the cell was much more significant, optimization or default value were not necessary. The results for RPq optimization process are displayed in Figure 1.

The precision data for the three sample matrixes are given in Table 3. The obtained results indicate acceptable precision in all cases, with an RSD of 4.22% for the water samples, and 2.46% and 3.06% for soil and plant samples, respectively.

The accuracy was estimated by comparing the certified and measured concentrations and by assessing the pooled recovery and confidence interval (CI) for the 95% confidence level (as illustrated in Table 4). Data for the analysis of Li concentration in CRMs of water, soil, and plant method showed recovery in the range of 92.9–111% and a pooled recovery of 96.2%.

### 3.2. Water Samples

**Figure 2:**
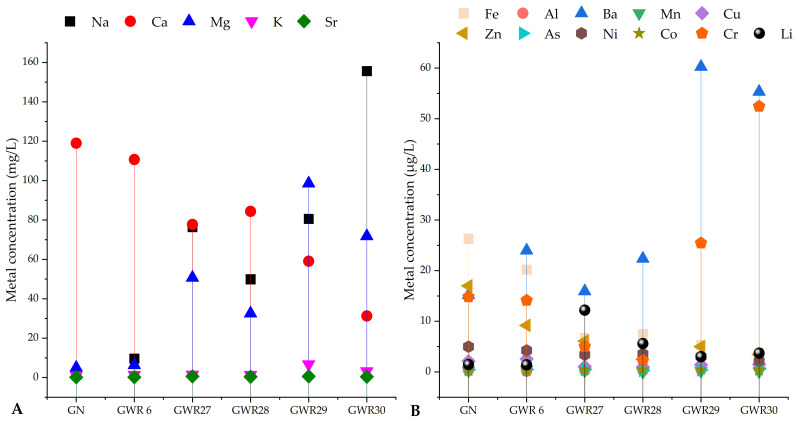
Water samples Li concentration (macro- (**A**) and microelement contents (**B**)) in comparison with other elements.

The Li concentrations in water samples in comparison with the macro- (Ca, K, Na, Mg, Sr) and microelements (Fe, Al, V, Cr, Mn, Co, Ni, Cu, Zn, As, and Ba) concentrations from the six sampling sites are shown in Figure 2 and in Supplementary Tables S1 and S2.

The highest Li concentration was measured in GWR27 followed by GWR28 (12.20 and 5.60 µg/L, respectively), while the lowest content was observed in GN and GWR6 (1.46 and 1.40 µg/L) sampling sites. The Li content in GWR27 was between 2 and 9-times higher than the other sites. More than 100-fold higher Na content was measured in water from GWR30 site than the water from GN site. Meanwhile, in the GN site, the Ca concentration was found to be higher compared to that of the other sites (from GWR27 to GWR30), except for water from GWR6. The highest Mg concentration was measured in waters of GWR29, which was 16 and 20-times greater than the Mg concentration in the water from the GN and GWR6 sites, respectively. The K concentrations in water samples were found to be similar, only in GWR29 exceeded 6 mg/L.

The concentrations of other elements, such as Al, Mn, As, Co Cd, Pb, and V, were low or in below the LOQ (lower than 0.70 µg/L) in all water samples. The highest Ba concentrations were observed in GWR29 and GWR30, almost 4 times higher than in waters from GN, GWR6, and GWR27. However, the highest Fe and Zn concentrations were measured in GN and GWR6. For Cr, the highest concentrations were found in the waters of GWR30 and GWR29. Interestingly, high Sr concentrations were obtained in the water samples from the GWR27 and GWR29 sites.

### 3.3. Soil Samples

**Figure 3:**
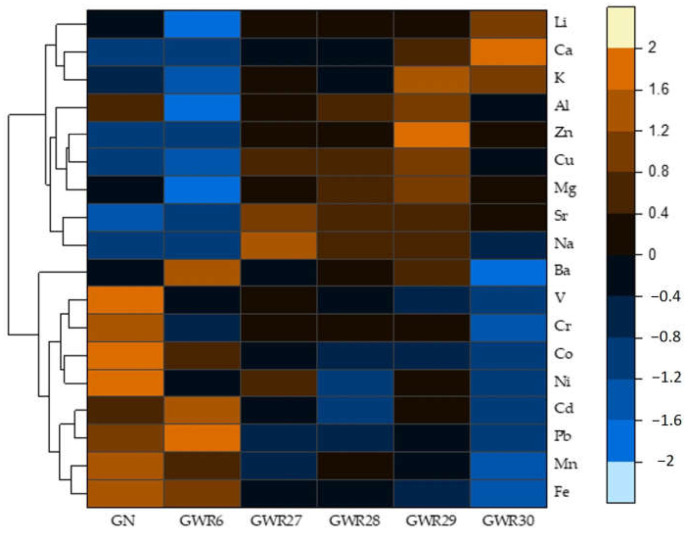
Logarithmic (Log10) values of Li in comparison with other macro- and microelements presented in heatmap with hierarchical clustering dendrogram of elements in soil samples from GN, GWR6, GWR27, GWR28, GWR29, and GWR30 sampling sites.

The distribution of elements in soil samples are presented in Figure 3 and in Supplementary Tables S1 and S2. Li content had no relevant spatial variation within the two study regions; however, some differences were observed. The soil samples from Dobrogea had a Li content ranging between 9.85 and 11.3 mg/kg DW, while in Banat, the Li content ranged from 6.50 to 9.95 mg/kg DW. The highest Li content was measured in the GWR29 site, almost 2-fold greater than the soil sample from GWR6, where the lowest Li content (6.50 mg/kg DW) was measured. The GWR29 soil sample have high concentrations of Ca, K and Mg. The GWR29 soil had the highest Ca content, followed by GWR30 (200 and 111 g/kg of Ca), which was about 21-fold greater than in GN and GWR6, indicating a major difference in the chemical processes that controls the soil chemistry in the two studied areas. However, compared to that of the other sampling points, the highest Mg, K, Al, Cu, and Zn content were measured in soil of GWR30. The GWR6 soil sample with the lowest Li content had also the highest Pb and Ba contents. Ni, Cr, and Co contents from the GN soil sample were the highest. The Sr content in soil was the highest in case of the GWR27, similar to the water samples. The same tendency was observed for the Fe contents, with the highest values in GN and GRW6.

In the present study, the correlations and similarities among the chemical indicators of the soil samples were established by HCA, as shown in Figure 3. The performed HCA grouped the chemical parameters into three clusters. One cluster included Li, Ca, and K, the second cluster included Al, Zn, Cu, Mg, Sr, and Na, while the third cluster contained Ba, V, and almost all heavy metals (Cr, Co, Ni, Cd, Pb, Mn, and Fe). Li, Ca, and K clustered closely together in the dendrogram, suggesting that those determinants are highly correlated.

### 3.4. Plant Samples

**Figure 4:**
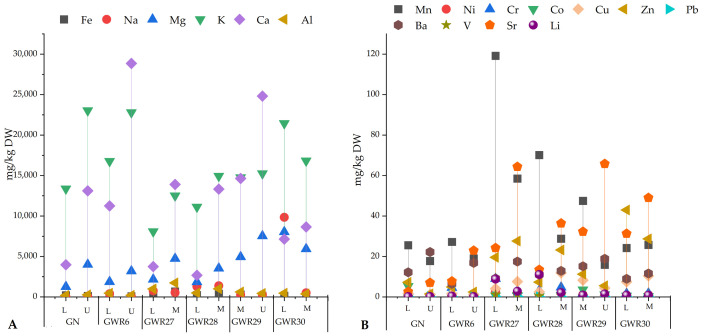
Macro (**A**) and microelement (**B**) content of plant samples (*Lolium* sp.—L, *Urtica* sp.—U, and *Mentha* sp.—M) in Banat (GN, GWR6) and Dobrogea (GWR27, GWR28, GWR29, and GWR30) region.

The highest Li content was measured in *Lolium* sp. collected from GWR28 and GWR27 (11.1 and 8.8 mg/kg DW) (as illustrated in Figure 4B). The *Lolium* sp. samples from GWR27 and GWR28 contained Li in 3- and 5-fold greater quantities than that of the *Mentha* sp. samples.

All the plant samples from GN and GWR6 had a low Li content (≤0.2 mg/kg DW, with the exception of *Lolium* sp. from GWR6 at 0.45 mg/kg DW). In GWR29 and GWR30, Li content of plants was about 1 mg/kg DW. The *Mentha* sp. sample in GWR27 had the highest Fe and Al content, while the *Lolium* sp. had the highest Mn content compared to that of the other plant samples in this study (as illustrated in Figure 4A). High Na, Mg, and K contents were observed in *Lolium* sp. from GWR30 (as illustrated in Figure 4A). The plants with a lower Li content had higher Ca and K and lower Mn and Fe contents (i.e., *Urtica* sp. from GWR6 and GN).

### 3.5. Data Analysis

The basic statistics (maximum, minimum, average, and standard deviation) for the microelements (Al, Mn, Ni, Cr, Co, Cu, Zn, Pb, V, Sr, and Li) in water, soil and plant samples in all the six-sampling locations are presented in Table 5.

**Figure 5:**
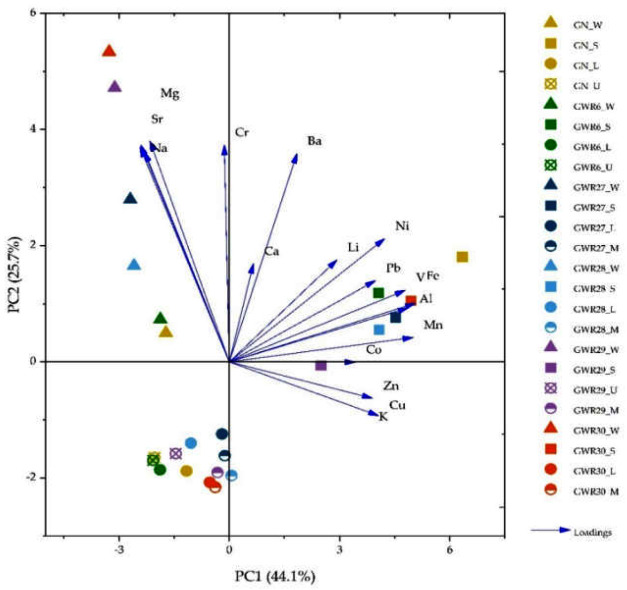
Principal component analysis (PCA) of variation of macro- and microelements in water, soil, and plant samples (L—*Lolium* sp., U—*Urtica* sp., and M—*Mentha* sp.) in Banat (GN, GWR6) and Dobrogea region (GWR27, GWR28, GWR29, and GWR30).

The PCA results in case of the macro and microelements are presented in the Figure 5. The obtained results indicate that first three principal components (PCs) explain 70% of total variance. PC1 with the highest total variance accounted for 44.1% of the total variation and showed the highest loadings for Fe, Al, Mn, and Ni, with notable loadings from Na, while PC2 revealed a 25.7% of the variability, with higher loadings for Na, Mg, Ba, Sr, Cr and with high contributions of K. Information about the correlations of macro- and microelements in water, soil, and plant samples are given through the position of the vectors. The PCA plot (as illustrated in Figure 5) shows the strong negative correlation between Na, Sr, Mg, and Li, stated by the position of the elements on opposite sides of the origin on the horizontal axis. A positive correlation between Ba and Ca or Na, Sr, and Mg can be seen by the same direction of the respective vectors; it can also be observed for Li, Ni, Pb, V, Fe, Al, Mn, Co, Zn, and K.

**Figure 6:**
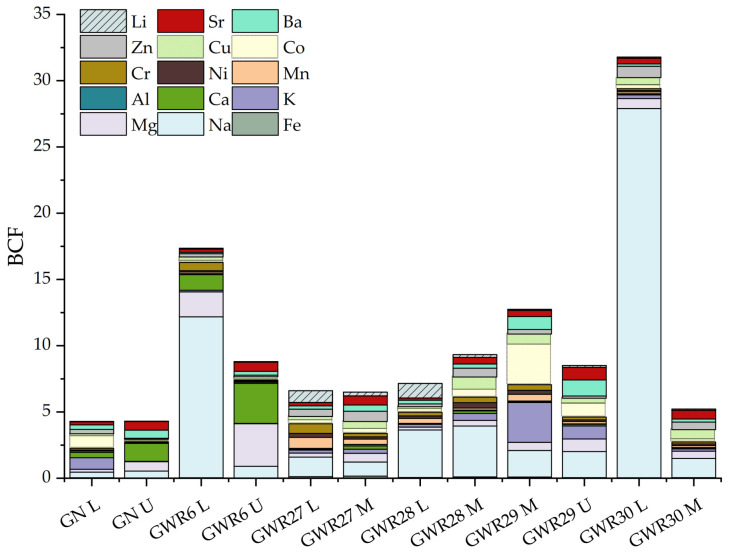
Bioconcentration factor (BCF) of macro- and microelements in plant species (*Lolium* sp.—L, *Urtica* sp.—U, and *Mentha* sp.—M) in Banat (GN, GWR6) and Dobrogea (GWR27, GWR28, GWR29, and GWR30) region.

Therefore, the plants BCF was calculated to determine the accumulation of Li and other macro- and microelements in the sampled plants from Banat and Dobrogea regions. According to the obtained results, the Li uptake behavior show the plants adaptation to specific soil conditions. The plants nutrient uptake is influenced by the top soil metal concentrations. High Na BCF factor was observed in case of the *Lolium* sp. from the GWR30 sampling point (as illustrated in Figure 6), followed by the *Lolium* sp. from the GWR6 location.

## 4. Discussion

Li concentration in the studied water samples differed among aquifer lithotypes because of the difference in Li abundance among rock types. Moreover, Lindsey et al. stated that aridity and aquifer age and its proximity to geothermal features are also definitory factors in controlling the evaluation of Li concentrations in water [4]. The highest concentration of Li in water was identified in GWR27 and GWR28, the sampling sites characterized by sandstone, while the lowest Li concentration was in GN and GWR6, characterized by areas with a limestone-confined aquifer. Similar results were reported in the United State, where carbonate rocks had low Li content, while unconsolidated sandstone and crystalline-rock aquifer had high Li content [4].

The chemical composition of water is also highly influenced by hydrology, water-rock interactions, and chemical weathering fluxes. Li is part of the alkali metal groups such as Na, which can act similarly during weathering processes according to Steinkoenig et al. [23]. The soil Li content is mainly controlled by weathering processes or atmospheric inputs and can be also correlated to water Li concentration. Generally, previous study showed that the Li content in soils was also correlated with Ca and K contents, which are derived from weathering of parental bedrock of transported alluvial or glacier materials [24]. Thus, the slightly higher Li content from waters and soils sampled from Dobrogea region could be due to the closeness with the Black Sea. Martin et al. estimated that, in the case of fresh water from Rottnest Island, atmospheric input via rainfall and sea-spray aerosols was the second most important source of Li, followed by carbonate dissolution [12,25].

The trace metal concentrations were in the admissible range for the Banat and Dobrogea regions according to the limit values established in Romanian legislation for water bodies and alert thresholds for sensitive soil uses [26,27].

The correlation of Li, Ca, and K content in soil samples was reported also by other studies; K and Li could have a common source as a result of rock-water interaction and water-soil transfer mechanisms [24,28]. Li, Ca, and K were decoupled from the heavy metals (Fe, Mn, Pb, Cd, Ni, Co, and Cr) in the HCA suggesting that the firsts behave quite differently and independent to each other (as illustrated in Figure 3).

Plant species have different metabolisms and the elements accumulation capacity is highly influenced by the growth medium. The Li bioavailability, uptake, and accumulation could be influenced by several factors related to soil (pH, moisture, metal content, etc.) [29]. Various metals at higher concentrations can cause serious health disorder in plants due to their nonbiodegradability, high bioaccumulation rate, and biotoxicity effects [30]. However, plants can develop and can adapt to extreme environmental conditions, due to their unique uptake mechanism, soil-root-shoot nutrient transfer mechanisms, and metal interaction with the available metal mixtures from the growing area [30]. Nonessential elements such as Cd and Pb can produce synergistic or antagonistic effects on the uptake and accumulation of K, Ca, Mg, Fe, and Mn [30].

In the present study, for the plants with high Na content, BCF ratio indicates high salinity tolerance ability. Generally, Li is considered a nonessential element for plant growth and development, and for halophyte species (plants that tolerate moderate to high salt concentrations in their substrate), Li plays an important role in their metabolism. Moreover, Li was reported previously by several studies to enhance plant productivity, yield, early maturation, and resistance to diseases [31,32]. A Li accumulation in soil is the result of ions release from rocks to clay and soil, where it can be fixed into organic matter or mineral oxides [29]. The plants Li level is directly correlated with the uptake of Fe, Ni, Co, Mn, Cu, Al, Pb, or Cd from the soil [33]. High Li uptake can occur in soils with equally high Na content such as soil with a natric horizon (also known as solonetz soils). In the present study, the obtained results indicated that plants Li uptake could be enhanced by Na, soil samples with the highest Na content were measured in GWR27 and GWR28, where also the highest Li BCF values were found in the *Lolium* sp. plants. However, despite the high Na and Li presence in GWR30 soil samples, a low Li BCF was observed in *Lolium* sp. This can be attributed to the plant’s adaptation mechanism and the specific environmental/soil conditions that include the presence of other elements such are Mg, K, and Ca. These three elements can produce antagonistic effects on Li and synergetic effects on the uptake of Na. For GWR29 and GWR30 soils the high Ca content (2–4-fold greater than in GWR27 and GWR28) inhibits and competes with the Li uptake. Studies based on the Li soil-atmosphere-biosphere exchange showed that Li content had specific effects on the uptake of other essential or trace elements [34]. The plant Li content was negatively correlated with Mg, Mn, and Mo, while a positive correlation was noticed with Ca, Fe, K, and Zn, indicating that Li changes the function of the essential element transporters [34]. The Li ion can have an adverse effect on the plant growth and interferes with the Ca metabolism [35]. In the present study the lowest BCF of Li was obtained in plants sampled in GN and GWR6 sites, while the highest K and Ca BCF ratio were obtained in *Urtica* sp. and *Lolium* sp. from GWR6, followed by the *Urtica* sp. from GN site. These results indicate that a competitive interaction with Li may occur during the adsorption of essential elements, such as Ca and K. The low BCF values of Li was accompanied by high Ba and Sr ratio in *Urtica* sp. and *Mentha* sp. from GWR29 sampling site, which indicates that soils higher K and Ca content may regulate and enhance the Ba and Sr uptake in the plants. However, further studies need to be done to fully understand the importance and/or inadequate role of Li and its competition with other trace elements during the nutrient uptake in the rhizosphere zone.

## 5. Conclusions

In this study, a versatile measurement method was optimized for the determination of Li from multiple matrixes (water, soil, and plant) using ICP-MS technique. The obtained results indicated an acceptable precision in all studied matrixes and a reproducibility of 2.46–4.22%. Higher Li concentrations were obtained both for water and soil in Dobrogea region compared to Banat region. Despite the high Li concentrations in soils, it was mostly unavailable for the plant’s uptake and bioaccumulation, as shown by the low bioconcentration values. The highest Li content was accumulated in *Lolium* sp. plants from two sites from the Dobrogea region, most probably due to the Li bioavailability, influenced by the soil characteristics and other trace element content such as alkali and alkali-earth elements (K, Na, Mg, Ca, Ba). The obtained results revealed that the Li content in soil was correlated with Ca and K, suggesting a strong association between their chemical behavior. The Li uptake and translocation in plants could be attributed to the monovalent cation in the presence of potential ligands in soil helping Li mobility and accumulation in the rhizosphere. The prolonged high Li concentrations effect is still unknown and further studies are needed to understand the Li transfer from soil to plants, identification of plants species which can accumulate Li from contaminated areas, and the main Li uptake/ translocation mechanism in different plant species.

## Figures and Tables

**Table 1:** Geology and geographic settings of studied samples.

	Banat		Dobrogea			
	GN	GWR6	GWR27	GWR28	GWR29	GWR30
Location	44°54′3.82″ N 21°46′29.86″ E	45°11′16.66″ N 21°51′16.07″ E	45° 0′53″ N 28°22′43″ E	45° 0′40″ N 28°28′10″ E	44° 2′31″ N 27°57′46″ E	43°59′21″ N 28° 0′29″ E
Altitude (m a.s.l.)	180	220	139	88	57	78
Soil	Rendzinas	Rendzinas	Chernozems	Chernozems, alluvial soils	Rendzinas	Alluvial soils
Geology	Limestone	Limestone	Sandstone, limestone	Sandstone, limestone	Limestone, marl limestone, clays, dolomites	Limestone, clays, diatomites
Predominant plant species	*Lolium* sp., *Urtica* sp.	*Lolium* sp., *Urtica* sp.	*Lolium* sp., *Mentha* sp.	*Lolium* sp., *Mentha* sp.	*Mentha* sp., *Urtica* sp.	*Lolium* sp., *Urtica* sp.

**Table 2:** Used ICP-MS parameters for optimization of Li determination.

Operating Condition	Setting
Nebulizer gas flow	0.92 L/min
Auxiliar gas flow	1.2
Plasma gas flow	15
ICP RF power	1350 Watts
Lens voltage	7.75 Volts
Analog stage voltage	−1850
Pulse stage voltage	1050
Scan Mode	Peak Hopping
Dwell Time per Amu (ms)	200
Integration Time (ms)	4000
Detector	Analog
Calibration Coefficient (R^2^)	0.99954–0.99997
No. of reading per sample	5

**Table 3:** Precision data (standard deviation-SD, and relative standard deviation-RSD), limit of detection (LOD), and limit of quantification (LOQ) for Li determination in water, soil, and plant sample matrixes using Elan DRC II ICP-MS spectrometer.

Matrix	Concentration	SD	RSD %	LOD	LOQ
Water	12.2 µg/L	0.51 µg/L	4.22	0.12 µg/L	0.39 µg/L
Soil	9.95 mg/kg DW	0.20 mg/kg	2.46	0.20 mg/kg	0.66 mg/kg
Plant	8.83 mg/kg DW	0.24 mg/kg	3.06	0.01 mg/kg	0.03 mg/kg

(*n* = 3 parallel measurements).

**Table 4:** Certified and measured concentration of Li in certified reference materials (CRMs).

CRM	Matrix	Certified Value ± U *	Found Value ± CI **
1643f NIST SRM	Water	16.6 ± 0.35 μg/L	16.3 ± 1.39
SQC001 NIST SRM	Soil	103 ± 6 mg/kg	95.7 ± 5.38
NIM-GWB 10019	Plant	0.115 ± 0.009 mg/kg	0.13 ± 0.01
Recovery range (%)	-	-	92.9–111
Pooled recovery (%)	-	-	96.2

* U = is the expanded uncertainty for 95% confidence level ** CI = is the confidence interval for 95% confidence level (*n* = 3).

**Table 5:** Maximum (Max), minimum (Min), average (Avg), and standard deviation (SD) of microelement (Al, Mn, Ni, Cr, Co, Cu, Zn, Pb, V, Sr, and Li) concentration in water soil and plants.

	Al	Mn	Ni	Cr	Co	Cu	Zn	Pb	Ba	V	Sr	Li
**Water (µg/L)**												
**Min**	0.14	<0.7	2.15	2.27	0.23	0.98	2.50	<0.7	15.2	<0.7	180	1.40
**Max**	0.70	0.45	5.00	52.4	0.45	2.56	17.0	<0.7	60.3	<0.7	613	12.2
**Avg**	0.37	0.21	3.57	19.0	0.36	1.60	7.20	<0.7	32.2	<0.7	403	4.56
**SD**	0.20	0.15	0.98	18.3	0.10	0.62	5.34	<0.7	20.2	<0.7	190	4.06
**Soil (mg/kg DW)**												
**Min**	6237	92.7	4.03	4.33	1.22	5.42	20.3	0.93	15.6	5.46	10.9	6.50
**Max**	18760	261	13.5	19.1	6.14	13.7	50.4	6.54	58.2	24.8	94.7	11.3
**Avg**	14638	179	8.02	11.2	3.00	10.3	32.7	2.95	39.4	12.4	59.3	9.45
**SD**	4440	58.6	3.42	5.06	1.84	3.19	10.8	2.22	14.5	6.58	31.1	1.60
**Plant—*Lolium* sp. (mg/kg DW)**												
**Min**	187	24.2	0.73	1.65	0.3	1.59	4.93	0.06	6.50	<0.02	2.63	0.02
**Max**	991	119	2.85	9.01	5.3	7.48	43	0.21	12.2	1.26	31.3	11.1
**Avg**	521	53.3	1.26	4.21	1.5	3.49	16.4	0.14	9.56	0.39	15.9	4.30
**SD**	291	41.6	0.90	2.88	2.2	2.45	15.9	0.07	2.18	0.53	11.8	5.24
**Plant—*Urtica* sp.** **(mg/kg DW)**												
**Min**	203	15.9	0.37	0.30	0.10	1.24	1.54	0.05	16.9	<0.02	7.0	0.19
**Max**	439	19.1	0.50	0.91	1.20	4.43	5.55	0.13	22.2	0.14	65.8	1.54
**Avg**	301	17.6	0.46	0.52	0.50	2.31	3.18	0.08	19.3	0.05	31.9	0.66
**SD**	123	1.60	0.07	0.34	0.61	1.83	2.10	0.04	2.7	0.08	30.4	0.77
**Plant—*Mentha* sp.** **(mg/kg DW)**												
**Min**	372	25.7	1.09	1.75	0.36	7.59	11.3	0.07	11.6	0.18	32.3	0.88
**Max**	1734	58.5	2.03	4.78	3.65	11.9	28.7	0.26	17.5	1.35	64.4	2.84
**Avg**	933	40.1	1.48	2.86	1.44	9.56	22.7	0.13	14.3	0.66	45.5	1.70
**SD**	595	15.6	0.42	1.42	1.49	1.99	7.99	0.08	2.59	0.51	14.4	0.96

## Data Availability

The data presented in this study are available on request from the corresponding author.

## Supplementary Materials

The following are available online at https://www.mdpi.com/article/10.3390/ma14144002/s1, Table S1: Concentration (average ± standard deviation, n = 3) of macroelements in water, soil and plant samples (L = *Lolium* sp., U = *Urtica* sp., M = *Mentha* sp.). Table S2: Concentration (average ± standard deviation, n = 3) of microelements in water, soil and plant samples (*Lolium* sp., U = *Urtica* sp., M = *Mentha* sp.).
