# Supplementary material for: Assessment of Lithium, Macro- and Microelements in Water, Soil and Plant Samples from Karst Areas in Romania
Source: Materials (Basel). 2021 Jul 17;14(14):4002. doi: 10.3390/ma14144002 (PMC8306611; doi:10.3390/ma14144002)
Supplement: Supplementary file 1 [file materials-14-04002-s001.zip › materials-1275478-supplementary.pdf]

Supplementary Material

Assessment of lithium, macro- and microelements in water, soil and plant samples from karst areas in Romania

Anamaria Iulia Torok[1], Ana Moldovan[1,2*], Erika Andrea Levei[1], Oana Cadar[1], Claudiu Tănăselia[1] and Oana Teodora Moldovan[3]

[1] INCDO-INOE 2000, Research Institute for Analytical Instrumentation, 67 Donath Street, 400293 Cluj-Napoca, Romania; ana.moldovan@icia.ro (A.M.); erika.levei@icia.ro (E.A.L); oana.cadar@icia.ro (O.C.); claudiu.tanaselia@icia.ro (C.T); iulia.torok@icia.ro (I.A.T)

[2] Faculty of Materials and Environmental Engineering, Technical University, 103-105 Muncii Boulevard, 400641 Cluj-Napoca, Romania; ana.moldovan@icia.ro (A.M.)

[3] Cluj Department, Emil Racovita Institute of Speleology, 5 Clinicilor, 400006 Cluj-Napoca, Romania; oanamol35@gmail.com (O.A.M.)

* Correspondence: ana.moldovan@icia.ro

This online resource contains the following data:

The concentration of macro and microelements and standard deviation of the triplicates in water, soil and plant samples presented in Table S1 and Table S2, respectively.

Table S1. Concentration (average ± standard deviation, n=3) of macroelements in water, soil and plant samples (L=*Lolium* sp., U=*Urtica* sp., M=*Mentha* sp.).

Sample	Fe	Na	Mg	K	Ca
			**Water (µg/L)**		
**GN**	26.3 ± 1.84	1373 ± 68.6	5005 ± 551	1151 ± 104	119000 ± 11900
**GWR6**	20.2 ± 1.82	9598 ± 672	6314 ± 379	1370 ± 41.1	110700 ± 8856
**GWR27**	6.70 ± 0.54	76390 ± 5347	50730 ± 2537	1483 ± 104	77760 ± 8554
**GWR28**	7.50 ± 0.45	49890 ± 4490	32550 ± 977	1345 ± 121	84390 ± 3376
**GWR29**	5.30 ± 0.58	80600 ± 2418	98580 ± 6901	6726 ± 673	59060 ± 3544
**GWR30**	2.30 ± 0.09	155600 ± 14004	71790 ± 2872	3182 ± 350	31280 ± 1251
			**Soil (mg/kg DW)**		
**GN**	5640 ± 338	78.5 ± 7.06	5673 ± 454	2026 ± 60.8	9733 ± 681
**GWR6**	5390 ± 162	32.2 ± 1.61	992 ± 29.8	1345 ± 121	9537 ± 1049
**GWR27**	4347 ± 304	513 ± 35.9	7137 ± 785	2986 ± 209	57600 ± 6336
**GWR28**	4367 ± 131	360 ± 32.4	8310 ± 914	2451 ± 123	70767 ± 5661
**GWR29**	3094 ± 340	136 ± 6.82	7987 ± 479	4333 ± 217	199733 ± 15979
**GWR30**	3940 ± 315	353 ± 31.8	10780 ± 755	4480 ± 358	110933 ± 5547
			**Plant (mg/kg DW)**		
**GN L**	210 ± 18.9	32.9 ± 3.30	1256 ± 62.8	13375 ± 1070	3974 ± 318
**GN U**	123 ± 11.1	40.6 ± 3.66	4001 ± 240	23038 ± 2304	13113 ± 656
**GWR6 L**	206 ± 8.23	391 ± 19.5	1866 ± 112	16775 ± 1174	11249 ± 562
**GWR6 U**	82.9 ± 5.80	28.4 ± 2.56	3190 ± 351	22800 ± 684	28850 ± 2020
**GWR27 L**	515 ± 56.6	757 ± 68.1	2170 ± 217	8084 ± 485	3751 ± 150
**GWR27 M**	680 ± 408	546 ± 38.2	4732 ± 237	12513 ± 500	13900 ± 973
**GWR28 L**	220 ± 19.8	1290 ± 51.6	1855 ± 204	11129 ± 1113	2674 ± 134
**GWR28 M**	406 ± 24.3	1383 ± 82.9	3535 ± 106	14938 ± 896	13325 ± 800
**GWR29 M**	281 ± 19.6	273 ± 21.8	4940 ± 445	14763 ± 1476	14638 ± 439
**GWR29 U**	141 ± 7.06	269 ± 10.8	7533 ± 452	15250 ± 1525	24813 ± 2481
**GWR30 L**	141 ± 15.5	9838 ± 984	8024 ± 401	21450 ± 2145	7145 ± 357
**GWR30 M**	174 ± 12.1	510 ± 51.0	5933 ± 297	16825 ± 1682	8649 ± 865

Table S2. Concentration (average ± standard deviation, n=3) of microelements in water, soil and plant samples (L=*Lolium* sp., U=*Urtica* sp., M=*Mentha* sp.).

Sample	Al	Mn	Ni	Cr	Co	Cu	Zn	Pb	Ba	V	Sr	Li
						**Water (µg/L)**						
**GN**	<0.7	<0.7	5.00	14.8	<0.7	2.12	17.0	<0.7	15.2	<0.7	180	1.46
	ND	ND	± 0.20	± 1.33	ND	± 0.06	±1.87	ND	± 1.67	ND	± 16.2	± 0.07
**GWR6**	<0.7	<0.7	4.25	14.1	<0.7	2.56	9.19	<0.7	24.0	<0.7	194	1.40
	ND	ND	± 0.26	± 0.85	ND	± 0.28	± 0.28	ND	± 2.16	ND	± 15.5	± 0.08
**GWR27**	<0.7	<0.7	3.40	5.02	<0.7	1.06	6.10	<0.7	15.9	<0.7	613	12.2
	ND	ND	± 0.20	± 0.20	ND	± 0.11	± 0.49	ND	± 1.43	ND	± 49.0	± 0.73
**GWR28**	<0.7	<0.7	3.58	2.27	<0.7	0.98	2.50	<0.7	22.4	<0.7	399	5.60
	ND	ND	± 0.14	± 0.14	ND	± 0.04	± 0.20	ND	± 1.57	ND	± 43.8	0.28
**GWR29**	<0.7	<0.7	3.03	25.4	<0.7	1.40	5.00	<0.7	60.2	<0.7	609	3.00
	ND	ND	± 0.15	± 0.76	ND	± 0.15	± 0.15	ND	3.01	ND	± 36.5	± 0.24
**GWR30**	<0.7	<0.7	2.15	52.4	<0.7	1.47	3.40	<0.7	55.3	<0.7	425	3.70
	ND	ND	± 0.17	± 5.24	ND	± 0.09	± 0.31	ND	± 1.66	ND	± 34.0	± 0.15
						**Soil (mg/kg DW)**						
**GN**	17143	261	13.5	19.1	6.14	7.69	22.5	4.83	35.9	24.7	10.9	9.12
	± 1200	± 28.7	± 0.4	± 0.57	± 0.31	± 0.46	± 2.47	± 0.43	± 1.44	± 2.48	± 0.33	± 0.64
**GWR6**	6237	219	7.59	7.30	4.06	5.42	20.3	6.54	58.2	10.0	32.8	6.50
	± 499	± 24.1	± 0.46	± 0.73	± 0.20	± 0.60	± 1.22	± 0.52	±5.82	± 0.90	± 2.95	± 0.26
**GWR27**	15283	143	9.70	12.5	2.87	12.6	35.7	1.64	36.7	13.3	94.7	9.95
	± 1223	14.3	± 0.58	± 1.37	± 0.26	± 0.88	± 3.21	± 0.11	±2.57	± 0.40	± 8.52	± 1.00
**GWR28**	16553	183	4.97	11.6	1.63	12.0	34.7	1.68	40.4	11.5	74.7	10.0
	± 828	± 14.6	± 0.45	± 0.46	± 0.13	± 0.72	± 1.74	± 0.08	± 2.43	± 0.80	± 3.73	± 0.50
**GWR29**	13853	92.7	4.03	4.33	1.22	10.1	32.8	0.932	15.6	5.46	69.3	11.3
	± 1247	± 7.41	± 0.24	± 0.35	± 0.05	± 0.60	± 2.30	± 0.04	± 0.62	± 0.27	± 7.63	± 0.79
**GWR30**	18760	173	8.33	12.23	2.08	13.7 ±	50.4 ±	2.07	49.8	9.52	73.3	9.85
	± 2064	± 6.93	± 0.42	± 0.98	± 0.08	0.82	5.04	± 0.08	± 4.48	± 0.67	± 4.40	± 0.69
						**Plant (mg/kg DW)**						
**GN L**	187	25.6	0.80	2.64	5.35	1.74	7.26	0.16	12.2	<0.02	2.63	0.21
	± 13.1	± 2.82	± 0.06	± 0.21	± 0.16	± 0.12	± 0.29	± 0.01	± 0.85	ND	± 0.26	± 0.02
**GN U**	259	17.8	0.37	0.30	0.20	1.24	1.54	0.13	22.2	<0.02	7.04	0.24
	± 28.5	± 0.53	± 0.01	± 0.03	± 0.01	± 0.11	± 0.05	± 0.01	± 0.89	ND	± 0.42	± 0.03
**GWR6 L**	466	27.2	0.73	4.56	0.50	1.59	4.93	0.21	6.50	<0.02	7.78	0.45
	± 41.9	± 2.72	± 0.07	± 0.50	± 0.02	± 0.18	± 0.54	± 0.02	± 0.39	ND	± 0.23	± 0.02
**GWR6 U**	203	19.1	0.49	0.36	0.10	1.27	2.45	0.06	16.8	<0.02	22.9	0.19
	± 12.2	± 0.57	± 0.03	± 0.01	± 0.01	± 0.08	± 0.27	± 0.01	± 1.69	ND	± 0.92	± 0.01
**GWR27 L**	991	119	2.85	9.01	0.65	4.14	19.6	0.20	9.13	1.26	24.2	8.83
	± 59.5	± 10.7	± 0.20	± 0.54	± 0.05	± 0.41	± 1.96	± 0.01	± 0.64	± 0.13	± 1.94	± 0.88
**GWR27 M**	1734	58.5	1.60	3.10	0.89	7.59	27.6	0.26	17.5	1.35	64.4	2.84
	± 156	± 4.10	± 0.05	± 0.09	±.044	± 0.23	± 2.76	± 0.02	± 1.40	± 0.09	± 5.79	± 0.11
**GWR28 L**	493 ±	70.2	0.89	3.20	0.34	2.51	7.34	0.08	11.4	0.53	13.5	11.1
	54.3	± 3.51	± 0.03	± 0.10	± 0.03	± 0.10	± 0.81	± 0.01	± 0.77	± 0.06	± 1.48	± 0.89
**GWR28 M**	1010	28.8	2.03	4.78	0.86	11.9	23.2	0.12	12.9	0.71	36.4	2.17
	± 70.7	± 3.16	± 0.12	± 0.29	± 0.07	± 1.31	± 1.16	± 0.01	± 1.16	± 0.05	± 3.64	± 0.17
**GWR29 M**	618	47.5	1.20	1.83	3.65	8.29	11.3	0.09	15.2	0.40	32.3	0.93
	± 61.7	± 1.9	± 0.07	± 0.07	± 0.26	± 0.41	± 0.90	± 0.01	± 1.06	± 0.04	± 0.97	± 0.03
**GWR29 U**	439	15.9	0.50	0.91	1.20	4.43	5.55	0.05	18.9	0.14	65.8	1.54
	± 21.9	± 1.27	± 0.05	± 0.10	± 0.06	± 0.35	± 0.61	± 0.00	± 1.13	± 0.01	± 3.95	± 0.14
**GWR30 L**	467	24.2	1.02	1.65	0.58	7.48	43.1	0.06	8.96	0.18	31.3	0.90
	± 51.4	± 2.18	± 0.03	± 0.13	± 0.05	± 0.75	± 3.87	± 0.01	± 0.81	± 0.01	± 1.25	± 0.10
**GWR30 M**	372	25.7	1.09	1.75	0.36	10.4	28.7	0.07	11.6	0.18	49.0	0.88
	± 33.5	± 1.54	± 0.11	± 0.11	± 0.03	± 0.31	± 3.16	± 0.01	± 0.93	± 0.01	± 3.43	± 0.07

*ND – not determined

Author Contributions: Conceptualization, I.A.T., E.A.L. and O.C.; methodology, I.A.T., A. M. and C.T.; analysis, I.A.T., A. M. and C.T.; writing—original draft preparation, I.A.T., A.M. and C.T.; writing—review and editing, O.C., E.A.L. and O.T.M.; funding acquisition, O.T.M. All authors have read and agreed to the published version of the manuscript.

Funding: Sampling and laboratory analysis were supported by a grant of the Ministry of Research, Innovation and Digitization, CNCS/CCCDI – UEFISCDI, project number 2/2019 (DARKFOOD), within PNCDI III, and results interpretation were supported by the EEA grants 2014-2021, under project GROUNDWATERISK, contract no. 4/2019. The APC was funded by the Ministry of Research, Innovation and Digitization, CNCS/CCCDI – UEFISCDI, project number 2/2019 (DARKFOOD), within PNCDI III.

Data Availability Statement: The data presented in this study is available on request from the corresponding author.

Institutional Review Board Statement: Not applicable.

Informed Consent Statement: Not applicable

Conflicts of Interest: The authors declare no conflict of interest. The funders had no role in the design of the study; in the collection, analyses, or interpretation of data; in the writing of the manuscript, or in the decision to publish the results.
